# Resident Operating Surgeons?

**Published:** 1921-04-09

**Authors:** 


### RESIDENT OPERATING SURGEONS?

As previously adumbrated in our columns, something has been heard of a proposal to do away with the time-honoured hospital system of honorary and resident (as regards surgery, at least), and to substitute in non-teaching institutions a paid resident operator, to whom would fall the entire treatment of all surgical cases admitted. The arguments advanced in support of the proposal, which came from a lay source, were mainly as follows: It was urged that accident and urgent cases, frequent in the industrial area concerned, were often kept waiting for definitive diagnosis and treatment through the absence of the senior surgeon, who was attending to the claims of his private practice. Furthermore, the patient suffered additionally in that the optimum therapeutic measures were not applied at once, as also because not seldom the pain of re-examination, redressing, and so forth was incurred through the senior having to satisfy himself as to the details of the case after his junior had already done so. The actual motion was for the engagement of an F.R.C.S. (England) at a salary of £1,000 a year, to live in, or in immediate proximity to, the hospital, and to be solely responsible for all surgical cases admitted thereto. The mover promised that the increased expense would be met by increased working-men's contributions, and he contemplated the prospect of the resignation of the existing honorary staff with equanimity. The hospital in question has a medical as well as a surgical side; the physicians, though not feeling their withers wrung so much as their surgical colleagues did, yet intimated informally and in friendly fashion that they would show solidarity with them in the matter.

From this stage the affair has now advanced another step. On the whole the result has been a gain to the motion; for it has enlisted the support of lay representatives other than labour ones. Certain employers—as, for example (to take an instance quite removed from the area in question), the London and North Western Railway Company at Crewe—have already special hospitals staffed as above described, the patients in which are solely employees suffering from injury or its sequelæ. Such employers would be likely to be, and have been, impressed by the course suggested. We do not ourselves pass an opinion thereon. It is interesting, however, to consider the changes which anything like a wide adoption of it would mean to medical life. To begin with, it is hardly likely that the arrangement in question would meet with universal professional opposition, at all events just now. As is well known (and the fact will be brought before committees in discussions of this nature), the British Medical Association has urged hospital "honoraries" to apply for a share of the fees payable for institutional treatment of ex-Service men. Such claims must, of course, somewhat weaken the honorary status of the claimers. Then at the present time there are a great number of young surgeons to whom the war has given operating experience far beyond their years, while at the same time depriving them of the ordinary chances of civil advancement. These gentlemen would be very well suited indeed by posts such as the proposal under notice would create all over the country. Further, the tendency of the day is to increase salaried medical work and decrease private practice. The claims, on the other hand, of vested interests to protection are too obvious to need mention for the moment. However, we only pose the question here; we do not profess to give any definite verdict. The opinions of our readers would be very interesting, and we hope to be favoured with some.

